# Impacts of Transitioning to an Online Curriculum at a Graduate School in South Korea Due to the COVID-19 Pandemic

**DOI:** 10.3390/ijerph191710847

**Published:** 2022-08-31

**Authors:** Eric Yee, Changhwa Jung, Derrick Cheriberi, Minjune Choi, Wonsick Park

**Affiliations:** KEPCO International Nuclear Graduate School, Ulsan 45014, Korea

**Keywords:** online classes, COVID-19, graduate school, isolation

## Abstract

This study focuses on the impacts of implementing an online curriculum at a graduate school in South Korea in response to the COVID-19 pandemic. A framework distinguishing impacts to academic, educational, and institutional stakeholders from the virtualization of curricula as well as general COVID-19 prevention measures is invoked to help understand the impacts of these changes. These impacts are sourced from general graduate school operations, course evaluations for two compulsory courses, and unofficial interviews with students and professors. A statistical evaluation of the course evaluations suggested no significant difference between the online format of 2020 and the traditional in person formats in prior years in terms of academics and education. Unofficial meetings with students and faculty revealed technical issues throughout 2020, which many could not be resolved due to the variety of different computer systems at the school as well as limited technical support. Most importantly, students stated they were suffering from prolonged mental and emotional distress such as feeling isolated. Lessons learned include having academic institutions prepare for difficulties in technical support, educational infrastructure investments, compliance, as well as student body mental health.

## 1. Introduction

The COVID-19 pandemic continues to have a strong impact on modern civilization, from public health and global economics to the more local labor and education sectors. The education sector’s response to COVID-19 has varied from region to region, but many schools and universities have implemented or considered implementing online versions of their curriculum. Having an online curriculum minimizes the time students and instructors spend in the presence of others and therefore minimizes the spread of COVID-19. However, transitioning to an online format has led to some negative reactions from stakeholders in the learning process. Many students in higher education feel the costs of tuition are not on par with an online academic experience along with a lack of networking opportunities. Similarly, some instructors feel virtual classrooms are less efficient academically and therefore result in lower quality. Moreover, some academic institutions feel unprepared for such a change. Interestingly, parents of younger students argue an online curriculum of virtual classrooms creates additional professional, financial, and social burdens for families [1,2,3].

There is a fair amount of literature on the integration and utilization of an online format in school curricula. Several studies describe a school’s sudden implementation of an online curriculum in response to crises and natural disasters, such as hurricanes [4], earthquakes [5,6,7], the 2002 Severe Acute Respiratory Syndrome, SARS outbreak [8,9], the 2015 Middle East Respiratory Syndrome, MERS outbreak [10], and the current COVID-19 pandemic [11,12,13,14,15,16,17,18,19,20,21,22]. Insights from these studies say a strong telecommunications and information technology infrastructure is needed to properly benefit from online learning technologies as well as a strong impact on students’ mental health from COVID-19 protocols.

Additionally, online learning technologies have been well studied over the years. One study provides a good literature review on a variety of technologies applied to the classroom and found many studies treated positive student appraisals as evidence of academic benefits as opposed to realizing academic objectives [23]. Another study provides an academic comparison between traditional and online classrooms and suggests the differences in performance are not related to modality, but on certain personal factors [24]. These factors appear in other studies and seem to depend on how motivated students are [17,23,25,26,27]. Conversely, another study suggests academic effectiveness is tied to instructional design factors such as modality (e.g., fully online, hybrid of online and offline), synchrony (e.g., synchronous or asynchronous interaction), and the instructor’s role online [28]. 

Given this backdrop, this study attempts to elucidate how stakeholders of a medium sized graduate school in South Korea are impacted by the transition to an online curriculum. Findings from literature will be used as guidance in developing a simple framework to help understand the impact COVID-19 had on graduate students. This framework will be applied to course evaluation surveys, personal interviews, and relevant administrative work by the first author. One unique aspect of this study is that the telecommunications infrastructure in South Korea is well-developed and that virtually all graduate students were on a scholarship, thus minimizing telecommunications infrastructure and financial concerns from COVID-19 effects on academics and mental health respectively; relationships previous works had a hard time decoupling [4,5,6,7,8,9,18,19,20,29,30]. Hopefully, the results will add to an important database that monitors the experience of students and schools in their transition to online learning as well as valuable guidance on how to utilize online learning to better serve students and practitioners.

## 2. Materials and Methods

The graduate school under study, hereafter noted as Graduate School K, is located along the southeastern coast of South Korea, in a relatively rural area. Every year, the student body mix at Graduate School K is equally split amongst domestic and international origins. Most have considerable work experience and virtually all are on scholarship covering school-related expenses. Some input from the first author, who served as Head of the Department of Nuclear Power Plant, NPP, Engineering at Graduate School K is also provided to add some perspective. 

To better understand the impacts of transitioning to an online curriculum, a description of the stakeholders is needed. In this study, the primary stakeholders are the participants in the learning process and are abstractly designated as (1) students: the individuals that benefit from lessons and experiences provided, (2) instructors: the individuals that provide lessons, and (3) institutional: the individuals, organizations, and systems that link students and instructors and provide the infrastructure for doing so. For consistency and ease-of-use, the role of students, instructors, and institutions will be hereafter termed Academic, Educational, and Institutional, respectively. Impacts to primary stakeholders will be described by any action, reaction, activation, or implementation related to online instruction or COVID-19 matters. Guidance on how to evaluate such impacts upon a stakeholder is provided in the literature.

The literature suggests Academic stakeholders are susceptible to impacts involving health, sociology, and achievement [14,15,16,17,18,19,20,21,31,32,33,34,35]. The generalization of Health as being the overall physical, mental, and emotional condition with regards to academic activities relevant to the individual. The generalization of Sociological as being the interactions and relationships a student would be expected to have with their academic environment. The influence of finances would be categorized as a Sociological impact as it is not an issue that originates from the individual (i.e., [18,19,20]). The generalization of Achievement as being the results of general academic efforts, typically related to academic motivation and cognitive engagement. For brevity, examples of such instances are listed Table 1. 

The literature also provides guidance on impacts to Educational stakeholders, such as subject mastery and pedagogy [14,22,36,37,38,39,40,41,42,43]. The generalization of Subject Mastery relates to the inherent knowledge and experience that the lecturer has while the generalization of Pedagogy is related to the abilities to instill knowledge to academic stakeholders. It is commonly argued that instructors with good subject knowledge, but ineffective teaching abilities, have not been able to provide proper instruction. Conversely, instructors with effective communication techniques, but without subject knowledge mastery, may convey false or inadequate knowledge i.e., [38]. Interestingly, issues such as student engagement, positive teacher-student relationships, and expectations are also categorized as Academic, that is student-oriented as opposed to instructor-oriented. Admittedly, the distinction is a gray area, but generally the graduate school experience suggests most teacher-student interactions to be initiated by students than instructors. The idea is that graduate school is not a mandatory experience and graduate students participate of their own volition. On the more social aspect, classroom management, sometimes tied with student discipline, is also listed as a beneficial skill for instructors. However, student discipline is not considered an Educational impact herein, with many if not all manifestations, being captured as an Academic impact. Access to educational resources will also be considered belonging to Pedagogy as it is independent of Subject Mastery and influences an instructor’s teaching capabilities. For example, an instructor without a web camera in a synchronous online program would be extremely ineffective. Table 2 lists examples of impacts Educational stakeholders are exposed to.

However, the guidance for Institutional stakeholders is not as well-studied. Some studies suggest academic institutions are subject to risks related to business models, operating models, reputation, enrollment supply, and compliance [14,22,44]. Business model risks involve the institution’s ability to generate revenue such as through tuition, endowments, recruiting, education delivery, and financial controls. This is somewhat related to their description of operating model risks which are described as the ineffective processes, people, and systems that can negatively impact the institution’s core functions [44]. Enrollment supply is a steady supply of clients, in this case students, such that proper planning and funding can be implemented. Reputation entails the perception of the services and products of the academic institution, such as brand management, campus safety, and student activism. Compliance is the ability to adhere to relevant local and regional regulations and standards-of-practice, generally associated with national regulations, local regulations, research expenditures, and fraud. In this study, the generalization of business models and operation models are consolidated under Operations as shown with examples in Table 3. 

This framework is applied to impacts, previously defined as any action, reaction, activation, or implementation related to online instruction or COVID-19 matters, which includes results derived from administrative communications by the Head of the NPP Engineering Department with primary stakeholders as well as student course evaluations. Impacts to Academic, Educational, and Institutional stakeholders are tallied over the course of 2020 to help ascertain which stakeholders are affected most by online learning and COVID-19. Judgement is used in categorizing impacts to appropriate stakeholders. As an example, consider the situation of significantly changing school tuition. This would impact Institutional stakeholders as changing tuition would affect operations (revenue), enrollment supply (rising student debt), and reputation (brand management) as described in Table 3. Note that in this framework, Academic and Educational stakeholders are not significantly impacted as an increase in tuition does not directly affect health, sociology, or achievements of students as well as subject mastery or pedagogy from instructors. 

Special emphasis is placed on the student course evaluations as these provide historical data to compare conditions before and after the transition. Course evaluations consist of 12 base statements and are typically administered to participating students after completing the corresponding course. These evaluations have been conducted via online surveys through a vendor since 2016. These surveys presented a variety of statements, asking students to respond according to a Likert-type scale from 1 to 5, indicating “strongly disagree” to “strongly agree”, respectively. To give a comprehensive yet succinct picture on the students’ responses, a subset of the survey statements will be considered here. These statements are separated into two groups: Academic and Educational. Statements regarding the Academic aspects of the course, that is those aspects related to students’ perspectives on learning, include (a) understanding the concepts and principles in the subject was important to me and (b) this course helped me understand the concepts and principles in the subject. Statements regarding the Educational aspects of the course, that is those aspects related to students’ perspectives on the lecturer’s teaching, include (c) the professor was well prepared for class and (d) the professor presented content effectively (speech clarity, pace, volume). A single factor Analysis of Variance, ANOVA, will be applied to these course evaluation responses to determine if there are significant differences across years. If there are differences, a Bonferroni post-hoc test, which is essentially a *t*-Test assuming unequal variances with a Bonferroni correction [45], is applied to compare which years are statistically similar to 2020, the year online classes were implemented. These statistical tests help examine if online instructions were beneficial to students.

## 3. Results

### 3.1. First Semester, 2020

Right before the start of the first semester of 2020, the Ministry of Education, MOE, recommended schools postpone the start of the first semester and to hold online classes instead. However, the MOE had limits on the number of credits students could take in online courses and exceeding these limits would render a degree invalid. Later into the semester, around April, the MOE would later offer guidelines on holding offline classes if there were improvements in the local COVID-19 situation. Given the situation, the Department of NPP Engineering decided to hold courses in a synchronously online format for the first semester of 2020.

During the inaugural online semester, several issues developed. One was that a few international students could not physically attend Graduate School K due to a variety of travel-related issues. The challenge with synchronous online classes when international students are in their home countries is regional time differences, where a local standard schedule could be 6, 12, and 18 h off in other regions. This led to several professors offering an asynchronous option for their courses.

Secondly, a few students had laptop hardware and software issues. Graduate School K provided laptops as well as personal computers to those with significant hardware and software issues. Another issue was that instructional materials were not appearing properly on some students’ screens, with most complaints regarding resolution. The technical support staff provided as many solutions as possible, but not all issues could be resolved during the semester. These technical difficulties also boiled over into the faculty, with several professors stating the virtual classroom software did not integrate well with their presentations and software demonstrations. Due to these instructional obstacles, several professors indicated they prefer the traditional live, in-class format better. 

**Figure 1:**
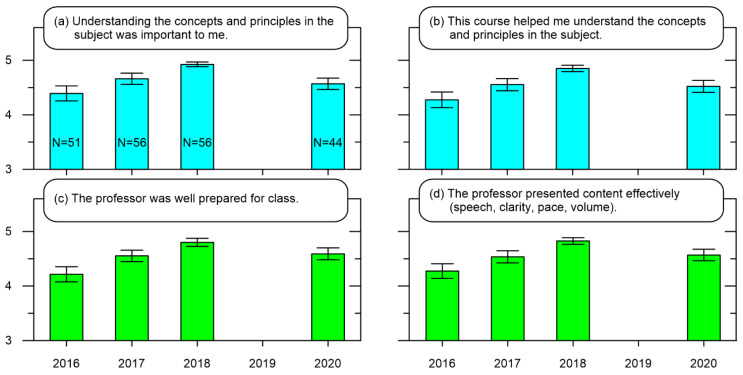
Compilation of course evaluation results for a 2020 first semester compulsory course at Graduate School K. Mean Likert-type scale results with 5 being “strongly agree” for academic-related statements: (**a**) understanding the concepts and principles in the subject was important to me, (**b**) this course helped me understand the concepts and principles in the subject, and education-related statements: (**c**) the professor was well prepared for class, and (**d**) the professor presented content effectively (speech clarity, pace, volume). There is a lack of data in 2019 because the course was taught by a different professor. N indicates the number of respondents for each year, which are the same for each survey statement presented.

Figure 1 presents a subset of the course evaluation results for the course “Introduction to Nuclear Engineering”. As mentioned previously, Figure 1a,b are related to the academic aspects of the course while Figure 1c,d are related to the Educational aspects of the course. Data from 2019 is not considered here because the instructor in charge for 2019 was different and so to maintain some consistency, the results from course taught by the same professor is shown. The results show the mean responses are between “agree” and “strongly agree” for each year, with a steady increase in the “strongly agree” responses. This is due to a reduction of “strongly disagree” and “disagree” responses from 2016 to 2018. The mean responses for 2020 show a slight decrease relative to 2018 as there were a sudden increase in “agree” and “neutral” responses relative to 2018. 

Applying single factor ANOVA with α = 0.05 to the data in Figure 1a,b reveals there are significant differences [F(3187) = 4.47, *p* = 0.005], [F(3187) = 3.87, *p* = 0.010] between the years. Interestingly, applying a Bonferroni post hoc test showed the responses from 2020 were not significantly different to those from the previous 2018, 2017, and 2016. Note the mean responses in 2018 were more positive than the mean responses from 2020. From an Academic perspective, these results suggest the differences between the online format of 2020 and live classrooms were not significant in terms of helping graduate students learn. 

Similarly, applying a single factor ANOVA with α = 0.05 reveals significant differences in the years for responses shown in Figure 1c [F(3187) = 3.93, *p* = 0.009] and Figure 1d [F(3187) = 3.87, *p* = 0.010]. Surprisingly, applying Bonferroni post hoc tests found no significant differences between 2020 against 2017 and 2016, but significant differences between 2020 and 2018. From an Educational perspective, these results suggest the differences between the online format of 2020 and live classrooms were not significant. The interpretation would be that the online format of 2020 was not an improvement in helping professors teach from a graduate student’s viewpoint.

Along with course evaluations, several unofficial interviews were conducted by the Head of the NPP Department with the faculty and students at the end of the semester. Virtually all faculty had issues with the online classroom software, where the program would freeze or did not integrate properly with other software. During the semester, technical support was unable to resolve these issues while software updates from the vendor resolved some issues. Another issue was web camera operations for synchronous online formats. A few students would not turn on their web cameras during virtual classes, saying that turning on the web camera slowed their laptop or smart phone down. Additionally, several students would log in to the system with their web cameras turned on, but then turn them off during the lecture. Interestingly, a few students said constant software malfunctions prevented them from properly logging on to the system. There were no known issues for asynchronous online portions.

In addition to curriculum changes, government guidelines recommended organizations reduce the worker density in office spaces. Graduate School K responded by having one person from each team work from home. This person would work from home for a pre-determined period of time and then switch with another colleague on the same team. The main issue was that not all school systems were available for off campus access as well as the limited online availability of support staff. Therefore, work from home policies and worker virtualization were less efficient in terms of administrative work.

An evaluation of the COVID-19 responses at Graduate School K during the first semester of 2020 is shown in Table 4. Travel restrictions for international impacted enrollment supply as potentially fewer students would apply to Graduate School K. Additionally, instructors had to account for international students that may arrive later in the semester, influencing teaching and academic objectives. The implementation of an online graduate curriculum introduced strains on all stakeholders. Graduate students had technical difficulties which primarily impacted Academic achievement. Instructors also had technical difficulties and had to modify instructional materials, impacting Educational pedagogy. Staff at Graduate School K had to support students and faculty while adhering to policies regarding online classes and social distancing. Moreover, a reduction in office manpower made school administration slightly less efficient.

### 3.2. Summer Session, 2020

For the summer session, traditional live, in-person classes were held. MOE released guidelines on how to hold live classes, which included students be at least 1 m apart, desks have dividers, all persons wear appropriate masks, and that all desks and chairs be wiped down after instruction. To follow these guidelines, the summer course had to split into multiple sessions as the school was unable to fit as many students in a classroom as before. Courses were able to post additional instructional materials online. Since the summer session course for 2020 was a new course, it is not used as a comparison herein.

At the end of the summer session, student representatives initiated several meetings with the President, the Dean of Academic Affairs, and Heads of departments over the conditions at Graduate School K. The student representatives were concerned with the mental health of the student body as many felt depressed and sad during and after the previous semester with continued COVID-19 restrictions and solely online instruction. Graduate School K did as much as they could to address graduate students’ concerns.

Table 5 lists the COVID-19 responses for the summer session of 2020. The student meetings revealed students were going through a difficult time. Although Graduate School K did not have medical personnel to identify and treat psychological disorders such as depression, the idea that student representatives would call a meeting to discuss such matters indicates the severity of the effects of COVID-19 prevention measures as well as the virtualization of the first semester. These effects will be characterized under Health and Sociological impacts as students described the first semester prevention measures as limits to social interactions students were normally allowed. These led to anger and frustration, which the student representatives said impacted their studies and put the school in a bad light, thus the inclusion of Academic achievement and Institutional reputation. The continuation of similar COVID-19 prevention measures into the summer only prolonged the stresses students felt. Additionally, adhering to national and regional COVID-19 prevention guidelines only increased the workload of an already confused and worrisome staff.

### 3.3. Second Semester, 2020

After the summer session, the MOE modified their policy regarding remote education, allowing up to 99% of credit hours being online. However, given the passionate criticisms from the students during the summer session and the relatively low number of new COVID-19 cases in the surrounding regions, Graduate School K prepared to hold courses live and in-person for the second semester. Unfortunately, the COVID-19 situation worsened right before the start of the second semester, forcing the school to re-evaluate the modes of instruction. In the end, the second semester was partitioned into continuous online and offline weeks, with compulsory courses being held a minimum of 6 weeks online.

**Figure 2:**
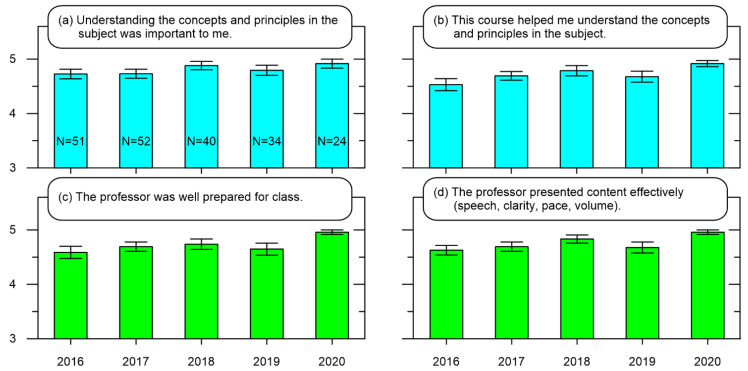
Compilation of course evaluation results for a 2020 second semester compulsory course at Graduate School K. Mean Likert-type scale results with 5 being “strongly agree” for academic-related statements: (**a**) understanding the concepts and principles in the subject was important to me, (**b**) this course helped me understand the concepts and principles in the subject, and education-related statements: (**c**) the professor was well prepared for class, and (**d**) the professor presented content effectively (speech clarity, pace, volume). N indicates the number of respondents for each year, which are the same for each survey statement presented.

Figure 2 presents the results that are most pertinent for a compulsory course, “Systems Engineering”. From 2016 to 2019, this course was taught using the local traditional method, but converted to a Flipped Learning format for 2020 [46,47]. Therefore, the initial portion of the course was conducted with online Flipped Learning and then proceeded to an in-class Flipped Learning approach. Although this implies an improper comparison, the examination of survey results should be viewed as a comparison between the hybrid online mode of instruction in 2020 against the traditional approach in previous years. The survey results are shown in a manner similar to Figure 1, where Figure 2a,b reveal the respondents’ academic conclusions on the course and Figure 2c,d provide insight into the quality of instruction. The results show the mean responses are close to “strongly agree” for each year, with a steady increase from 2016 to 2018, a slight drop in 2019, and then an all-time high in 2020. Again, applying single factor ANOVA with α = 0.05 revealed no significant differences for the data in Figure 2a [F(4198) = 1.44, *p* = 0.22], and Figure 2b [F(4198) = 1.94, *p* = 0.10]. Since there are no statistically significant differences between the years, Bonferroni post hoc tests are not necessary. From an Academic perspective, these results suggest perhaps one of two interpretations. One is that the course was designed in such a way as to be compatible with students’ academic interests at Graduate School K. Another is that the mode of instruction did not significantly affect the academic outcomes. 

Interestingly, applying single factor ANOVA to the data in Figure 2c,d with α = 0.05 revealed no significant differences [F(4198) = 0.90, *p* = 0.47] and no significant differences [F(4198) = 1.91, *p* = 0.11], respectively. As there are no significant differences, Bonferroni post hoc tests are not applied. Therefore, from an Educational perspective, these results suggest the differences between the hybrid online format and live classrooms were not significant. The interpretation would be that the hybrid format was not an improvement or detriment in helping professors teach from a graduate student’s viewpoint.

Similar to the end of the first semester, the Head of the NPP department conducted several unofficial interviews with the faculty and student body. The student body repeated the same concerns expressed at the end of the summer session, but did appreciate the Graduate School K’s attempts at mitigating them. Faculty continued to experience technical difficulties with the same online system. Several professors felt by the time in-person classes resumed, students were already behind in terms of material coverage. The issue of inactive webcams continued to come up and it appeared that more students were doing other activities while online classes were in session (e.g., picking up kids from school). Overall, students also seemed happy to resume live in-person instruction primarily for team assignment elements. Several students reported it was easier to organize and communicate in-person than online. 

Given responses and reactions to responses from online learning and COVID-19 prevention measures, Table 6 shows the impact on primary stakeholders. Similar to the first semester, utilizing the online learning software had similar outcomes. However, the resumption of live, in-person classes imparted significant changes. Firstly, the attention aspect of Academic achievement risk was minimized as the ability to discount instruction (i.e., deactivate webcam) was reduced in an in-person environment. Secondly, school staff had to adjust classrooms and perform monitoring activities to satisfy COVID-19 prevention related conditions for operating in-person classes. Many of these compliance and support activities also affected the annual budget. However, the COVID-19 prevention protocols continued to affect students’ mental health and interactions. Given prolonged adverse emotions amongst the student body, it is believed the school’s reputation was affected.

## 4. Discussion

To better understand how learning was influenced during 2020, Figure 3 shows the cumulative number of times COVID-19 prevention measures impacted Academic stakeholders. It shows a steady increase in instances where sociological matters were impacted, which is expected as both online learning and COVID-19 prevention measures would influence student interaction in one way or another. This is slightly surprising as student finances would be categorized as a Sociological impact, but even with full scholarships the category showed the highest sensitivity to COVID-19 responses. Moreover, COVID-19 response impacts to Health revolved around mental health, which appeared to affect Achievement, however these did not show up in a statistical analysis of the student course evaluation surveys. It should be noted the results regarding mental health were derived from communications with students by the Head of the NPP Engineering department and not as a part of the student course evaluations.

**Figure 4:**
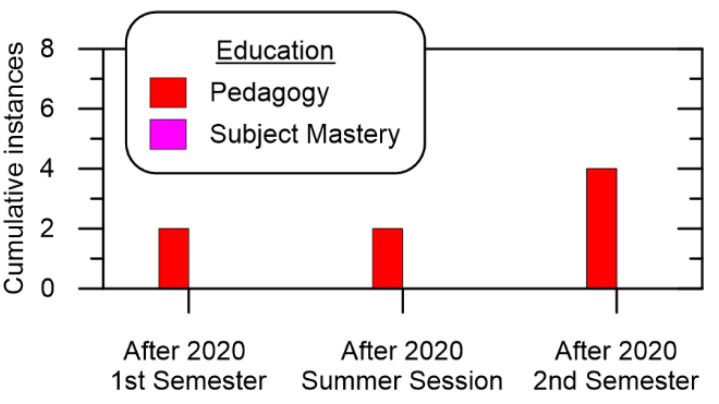
Cumulative Educational impact over time.

Conversely, Figure 4 shows the cumulative number of times COVID-19 prevention measures impacted Educational stakeholders. The figure shows in impact on instructors’ mastery of subject which is expected as both online learning and COVID-19 prevention should not influence an instructor’s knowledge base. Impacts to teaching were observed, especially in the first semester. This is due to adjusting to the online learning format, although it should be noted that most of the influence stemmed from the synchronous mode and not the asynchronous offerings.

**Figure 5:**
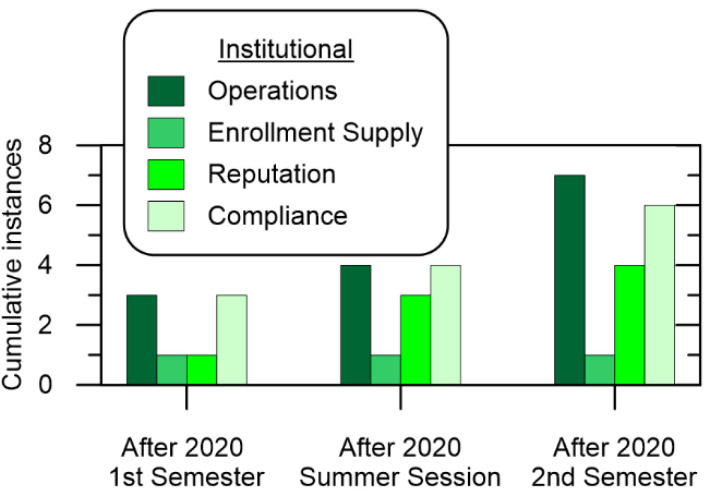
Cumulative Institutional impact over time.

Figure 5 shows the cumulative number of times COVID-19 prevention measures impacted Institutional stakeholders. Interestingly, there appeared to be a significant influence on operations and compliance. Surprisingly, the implementation of an online program would be the greatest challenge to compliance. MOE policies set in place were meant to deter schools from offering unconventional degrees, but in doing so, made it very difficult to make online curriculums practical. The MOE left several aspects open to interpretation, with some universities assuming the MOE would modify them before student graduations, which they did. Additionally, online learning put significant continuous strain on operations as technical support was constantly employed for both students and instructors. This was in part due to the variety of hardware and software systems in the academic environment. In addition to the technical aspects of online learning, one element of operations is the funding required for investments and expenditures. An additional ₩650,000 to ₩750,000 per student was spent to implement an online curriculum and to support COVID-19 prevention measures, most of which went to the online learning system implemented.

Overall, these findings are somewhat unexpected as a majority of the literature touts the benefits of transitioning to an online learning format with very little mention on the impact to institutions. For programs with limited support staff and budgets, careful preparation would be needed to tackle the technical and financial issues that will arise. Although a concern, the emergence of mental health issues in this study was similar to that found in several other recent studies, with slight differences in details depending on academic institution and region [14,17,18,19,20,21]. Interestingly, prior to such recent studies involving the influence of COVID-19 responses to the mental health of student bodies at academic institutions, much of the discussion focused on the benefits of online curricula.

## 5. Conclusions

A framework for understanding stakeholder impacts from transitioning to an online curriculum as well as COVID-19 prevention measures for the 2020 academic year was applied at Graduate School K. Academic and Educational reactions to the online learning format generally revolved around technical difficulties with a development of mental and emotional issues amongst the student body during the year. Even so, student course evaluations indicated no significant statistical difference overall between an online and offline format. The impact to Institutional stakeholders appeared the most, with activity in school operations and compliance to not only COVID-19 prevention policies, but in supporting the online curriculum implementation.

Taking these into consideration, the lessons learned from this online learning endeavor would be to: (1) prepare for additional technical issues, (2) expect an increase in educational technology expenditures, (3) prepare for an increase in compliance matters, and (4) prepare for additional mental and psychological stress in the student body. The first three can be addressed by the Institutional stakeholders, but the concerns with mental health within the student body is much more difficult to manage as conventional mitigation measures would involve Institutional stakeholders as well. Moreover, the study suggests implementing an online curriculum would have the academic institution incur additional strains. In this case of Graduate School K, the additional strains did not appear to lead to significant benefits for Academic and Educational stakeholders.

## Figures and Tables

**Figure 3:**
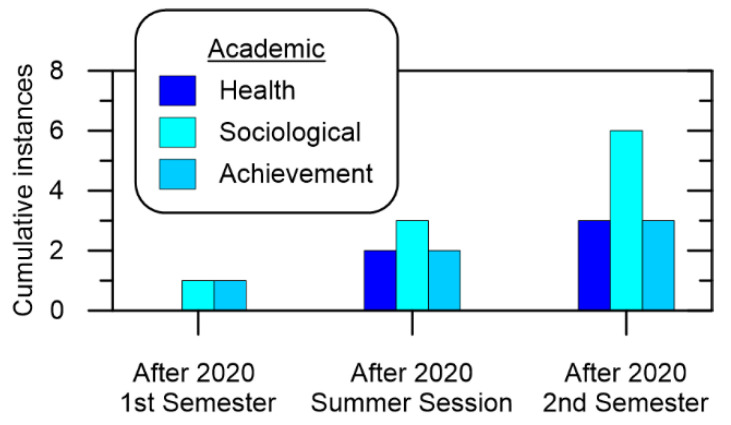
Cumulative Academic impact over time.

**Table 1:** Types of Academic impacts and examples.

Impact	Examples
Health	general health, physical health, mental health, psychiatric symptoms and disorders, substance abuse, behavioral issues
Sociological	social behavior amongst students and instructors, social integration, family issues, emotional support, dormitory life, extracurricular activities, socio-economic status
Achievement	grades, academic motivation, academic expectations, cognitive engagement, attendance/absenteeism

**Table 2:** Types of Educational impacts and examples.

Impact	Examples
Subject Mastery	subject knowledge, experience related to the subject, certifications, licenses
Pedagogy	positive teacher-student relationships, student engagement, classroom management, access to educational resources

**Table 3:** Types of Institutional impacts and examples.

Impact	Examples
Operations	revenue, recruiting, education delivery, financial controls, and processes, people, and systems that can impact the institution’s core functions
Enrollment Supply	immigration policies, growing economic markets, market demand, rising student debt
Reputation	brand management, campus safety, student activism
Compliance	national regulations, local regulations, research expenditures, fraud

**Table 4:** Impacts of the COVID-19 response for the first semester of 2020 at Graduate School K.

Response (March–June 2020)	Stakeholder Impact
Travel restrictions for international students	Educational: pedagogy Institutional: enrollment supply
Utilizing online learning tools	Academic: sociological Academic: achievement Education: pedagogy Institutional: operations Institutional: reputation Institutional: compliance
Inadequate computing tools for online learning	Education: pedagogy Institutional: operations
Work from home polices	Institutional: compliance Institutional: operations

**Table 5:** Impacts of the COVID-19 response for the summer session of 2020 at Graduate School K.

Response (July–August 2020)	Stakeholder Impact
Post-semester shock	Academic: health Academic: sociological Academic: achievement Institutional: reputation
Continued COVID-19 prevention measures	Academic: health Academic: sociological Institutional: operations Institutional: reputation Institutional: compliance

**Table 6:** Impacts of the COVID-19 response for the second semester of 2020 at Graduate School K.

Response (September–December 2020)	Stakeholder Impact
Utilizing online learning tools	Academic: sociological Academic: achievement Education: pedagogy Institutional: operations
Reverting to live, in-person classrooms	Academic: sociological Education: pedagogy Institutional: operations Institutional: compliance
Continued COVID-19 prevention measures	Academic: health Academic: sociological Institutional: operations Institutional: reputation Institutional: compliance
